# P2X7 Purinoceptor Affects Ectopic Calcification of Dystrophic Muscles

**DOI:** 10.3389/fphar.2022.935804

**Published:** 2022-07-14

**Authors:** Robin M. H. Rumney, Justyna Róg, Natalia Chira, Alexander P. Kao, Rasha Al-Khalidi, Dariusz C. Górecki

**Affiliations:** ^1^ School of Pharmacy and Biomedical Sciences, University of Portsmouth, Portsmouth, United Kingdom; ^2^ Department of Biochemistry, Laboratory of Cellular Metabolism, Nencki Institute of Experimental Biology, Polish Academy of Sciences, Warsaw, Poland; ^3^ Zeiss Global Centre, School of Mechanical and Design Engineering, University of Portsmouth, Portsmouth, United Kingdom; ^4^ Military Institute of Hygiene and Epidemiology, Warsaw, Poland

**Keywords:** P2X7 purinoceptor protects against ectopic calcification of dystrophic muscles duchenne muscular dystrophy, ectopic calcification, knockout, knockin, macrophage, P2X7

## Abstract

Ectopic calcification (EC) of myofibers is a pathological feature of muscle damage in Duchenne muscular dystrophy (DMD). Mineralisation of muscle tissue occurs concomitantly with macrophage infiltration, suggesting a link between ectopic mineral deposition and inflammation. One potential link is the P2X7 purinoceptor, a key trigger of inflammation, which is expressed on macrophages but also up-regulated in dystrophic muscle cells. To investigate the role of P2X7 in dystrophic calcification, we utilised the *Dmd* ^*mdx-*βgeo^ dystrophin-null mouse model of DMD crossed with a global P2X7 knockout (*P2rx7* ^*−/−*^) or with our novel P2X7 knockin-knockout mouse (*P2x7* ^*KiKo*^), which expresses P2X7 in macrophages but not muscle cells. Total loss of P2X7 increased EC, indicating that P2X7 overexpression is a protective mechanism against dystrophic mineralisation. Given that muscle-specific P2X7 ablation did not affect dystrophic EC, this underlined the role of P2X7 receptor expression on the inflammatory cells. Serum phosphate reflected dystrophic calcification, with the highest serum phosphate levels found in genotypes with the most ectopic mineral. To further investigate the underlying mechanisms, we measured phosphate release from cells *in vitro*, and found that dystrophic myoblasts released less phosphate than non-dystrophic cells. Treatment with P2X7 antagonists increased phosphate release from both dystrophic and control myoblasts indicating that muscle cells are a potential source of secreted phosphate while macrophages protect against ectopic mineralisation. Treatment of cells with high phosphate media engendered mineral deposition, which was decreased in the presence of the P2X7 agonist BzATP, particularly in cultures of dystrophic cells, further supporting a protective role for P2X7 against ectopic mineralisation in dystrophic muscle.

## Graphical Abstract

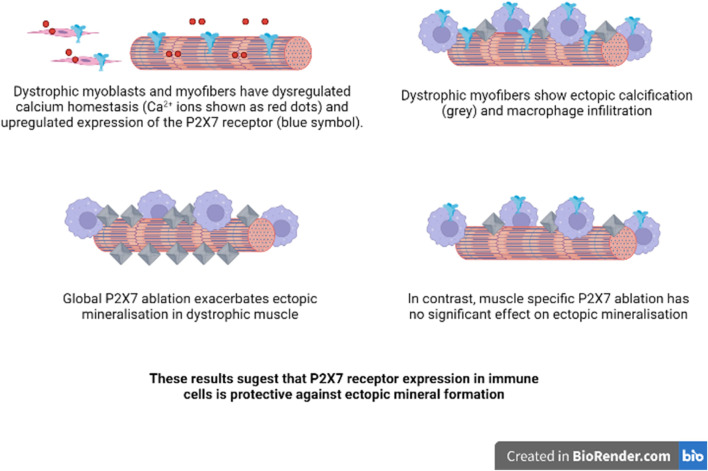

## 1 Introduction

Duchenne Muscular Dystrophy (DMD) is a devastating X-linked inherited disease, leading to severe disability and death in young men. Death is caused by the progressive degeneration of striated muscles aggravated by sterile inflammation (Sinadinos and Al-Khalidi, 2015). However, the pleiotropic effects of the mutant gene also include neuropsychiatric (Snow, Anderson, and Jakobson 2013) and bone structure abnormalities (Rufo et al., 2011), both irrespective of functional muscle impairment. These data indicate a much greater complexity of DMD pathology than the loss of structural integrity of muscle fibre sarcolemma. Indeed, while abnormalities of calcium homeostasis are widespread in DMD, its mechanisms, albeit not completely understood, have cell-specific components and can present in tissues structurally and functionally very dissimilar to muscle (Zabłocka, Górecki, and Zabłocki 2021).

Abnormally elevated resting cytosolic Ca^2+^ concentration is the common feature found across diverse cells affected by Duchenne muscular dystrophy (DMD) (Zabłocka, Górecki, and Zabłocki 2021). Recent studies revealed DMD pathology, including this calcium dys-homeostasis, to be active prior to diagnosis, with functional (van Dommelen et al., 2020) and molecular (Pescatori et al., 2007) abnormalities presenting in babies. In fact, studies in DMD foetuses and embryos from various animal disease models (Toop and Emery 1974; Emery 1977; Vassilopoulos and Emery 1977; Nguyen et al., 2002; Bassett et al., 2003; Merrick et al., 2009) revealed that this disease starts already in prenatal development, even before muscle specialisation (Mournetas et al., 2021). These developmental abnormalities are then recapitulated in myogenic cells in adult dystrophic muscle during their regeneration (Gosselin et al., 2021). However, once fully differentiated, myofibres appear to cope without dystrophin, as its ablation in mature skeletal muscle did not trigger their degeneration (Seno et al., 2008; Rader et al., 2016).

Furthermore, severe sterile inflammation is important in both the initiation (Pescatori et al., 2007; Y. W.; Chen et al., 2005; Haslett et al., 2002) and progression of the dystrophic phenotype (Lozanoska-Ochser et al., 2018; Peterson and Guttridge 2009). While an acute inflammatory response helps regeneration of damaged muscle (Tidball and Armando Villalta, 2010), chronic inflammation existing in DMD is detrimental (Tidball, Welc, and Wehling-Henricks 2018). Foci of inflammation generally precede irreversible muscle loss and fibrosis and are often associated with ectopic calcification (EC).

We observed recently that increased ectopic myofibre calcification is associated with altered macrophage infiltration patterns, particularly a close association of macrophages with calcified fibres (Young et al., 2020). Given that treatments reducing immune cell infiltrations significantly reduced the dystrophic pathology (Spencer et al., 2001; Farini et al., 2007; Evans et al., 2009; Boursereau et al., 2018; Sagheddu et al., 2018) and specifically that the more targeted reduction of inflammation *via* genetic ablation (Sinadinos and Al-Khalidi. 2015) or pharmacological inhibition (Al-Khalidi et al., 2018) of the P2X7 purinoceptor, the key inducer of the inflammatory response, produced a widespread functional attenuation of DMD symptoms (Górecki 2019), we investigated the role of this member of the P2X ATP gated ion channel family (Illes et al., 2021) in EC.

The association of P2X7 with inflammation and immunity is of critical importance (Adinolfi et al., 2018). P2X7 is involved in a range of responses: cytokine release, lymphocyte proliferation, intracellular pathogen killing, stimulation of gut mucosal immunity and even pain perception (reviewed in Di Virgilio et al., 2017; Young and Górecki 2018). P2X7 is expressed by virtually all cells of innate and adaptive immunity. Not surprisingly, P2X7 activation has been linked to a number of human diseases with an inflammatory component. Given that its overexpression was also found in dystrophic muscle cells (Yeung et al., 2006) and lymphocytes (Ferrari et al., 1994), we analysed the impact of both global and cell-specific ablation of the P2X7 purinoceptor on EC in dystrophic mice.

To facilitate this process, in this project we have used the dual purpose P2X7 knockin/knockout (P2X7^KiKo^) mouse. These mice were developed by us as a tool to better understand tissue-specific expression and function of this receptor, its subunit assembly, signalling pathways, molecular interactions, and functional regulation. While P2X7 receptor is an appealing therapeutic target, these vital data are still needed and mouse models are a very important tool in purinergic receptor studies (Rumney and Górecki 2020). To be able to explore the role of P2X7 in the variety of inflammatory and immune cells as well as in muscle, we generated mice expressing tandem-tagged receptors and with key exons flanked by *loxP* sites to allow identification of the native P2X7R composition and the full repertoire of its interacting partners by utilising the “genetic-proteomic” approach (Bienvenu et al., 2010) and also enabling tissue-specific ablation of this receptor by crossing this mouse with tissue-specific and/or conditional *Cre* recombinase expressing strains, leading to the excision of the *loxP*-flanked P2X7 region.

Using this approach allowed us to identify the tissue specific role of P2X7 receptor in EC of dystrophic mouse muscle. Understanding this alteration may lead to the development of new therapies for this debilitating disease but also for other pathologies where EC presents a real problem (Proudfoot 2019; Daniela, Federica, and Lofaro 2020).

## 2 Materials and Methods

### 2.1 Animals

Male C57BL/6-DmdGt (ROSAbgeo)1Mpd/J (*Dmd* ^*mdx-*βgeo^) and C57BL/6 eight week old mice were used in accordance with institutional Ethical Review Board and the Home Office (United Kingdom) Approvals. All mice were maintained under pathogen-free conditions and in a controlled environment (12-h light/dark cycle, 19–23°C ambient temperature, 45–65% humidity). For environmental enrichment, tubes, toys and nesting materials were routinely placed in cages. Dmd^m*dx-*βgeo^ mice were generated in C57BL/6 by insertion of the ROSAβgeo promoter-less gene trap construct downstream from the dystrophin DP71 promoter, resulting in the reading-frame disruption and loss of all dystrophin isoforms (Wertz and Martin Füchtbauer 1998) and *P2rX7* ^*−/−*^ knockout mice (Jackson lab s/n: 005576) were described before (Solle et al., 2001). The genotypes of all experimental animals were confirmed by PCR, as described before (Sinadinos and Al-Khalidi. 2015) (Specific primer sequences are summarised in Supplementary Table S1). Animals were killed by CO_2_ inhalation, tissues were dissected and used immediately for protein extraction, frozen in Liquid N_2_ or fixed with 10% buffered formalin for subsequent analyses. Blood samples were left to clot for 30 min prior to centrifugation at 2000g for 10 min to isolate serum.

### 2.2 RNA Extraction, cDNA Synthesis and qPCR Analysis

#### 2.2.1 RNA extraction and cDNA synthesis

Total RNA was extracted from tibialis anterior muscles using the RNEasy Plus Universal Mini kit (Qiagen 73404) according to kit manufacturer’s instructions. RNA quality and concentration were measured using a NanoDrop 1000 Spectrophotometer (Thermo Scientific). RNA integrity was assessed using electrophoresis of 100 ng of total RNA in a 1% agarose gel (Sigma A4718) in TAE buffer or using an automated capillary electrophoresis system (2100 Bioanalyzer Instrument G2939BA, Agilent) using a kit assay (Agilent RNA 6000 Nano Kit 5067-1511, Agilent). Total RNA samples were converted to cDNA using SuperScript VILO cDNA Synthesis Kit (Invitrogen 11754050) as per manufacturer instructions.

#### 2.2.2 Real Time Quantitative PCR

SYBR Green real-time qPCR reactions were ran as previously described (Sinadinos and Al-Khalidi. 2015). Based on our previous normalization of quantitative RT-PCR data using a set of 12 candidate reference genes (Primer Design) to ensure accurate quantification (Vandesompele et al., 2002), *Gapdh* was found amongst the most stably expressed genes (Sinadinos and Al-Khalidi, 2015). Therefore, it was used as reference to establish individual gene expression values (2^−ΔΔCT^). Amplifications were performed in duplicates using 25 ng of cDNA per reaction with Precision Plus Mastermix (Primer Design PPLUS-LR), forward and reverse primers (Supplementary Table S1) (Eurofins) and DEPC treated water (Fisher Bioreagents BP561) as per manufacturer instructions, on 96 well plates using an Applied Biosystem ViiA7 Real Time PCR instrument.

### 2.3 Cell Culture and Assays

#### 2.3.1 Isolation of Peritoneal Macrophages

Mice were killed by CO_2_ exposure, abdomens sterilised with 70% ethanol and 6 ml DMEM was injected into the peritoneal cavity. The peritoneum was then gently massaged and fluid aspirated. Collected peritoneal cells were centrifuged at 300 g for 8 min at 4°C. Supernatant was discarded and pelleted cells were re-suspended in 5 ml DMEM high glucose (HG) with 20% (v/v) FBS, 2 mM L- Glutamine, 100U/ml Penicillin and 100U/ml Streptomycin (Fisher Scientific, United Kingdom). Cell suspension was plated in polystyrene petri dish and incubated at 37°C in a humidified environment of 5% CO_2_ overnight. Non-adherent cells were then thoroughly rinsed after 2 h.

#### 2.3.2 Isolation of Bone-Marrow Derived Macrophages

Femora and tibiae were dissected and cleaned mechanically using paper tissue to remove the surrounding muscle tissue. Bones were placed in cold PBS in a petri dish and transferred to a sterile hood. After soaking for 30 s in 70% ethanol and two washes in sterile PBS, the contents of bone marrow cavities were flushed out with cold PBS using a syringe and a needle. Eluates were passed through a 70 µm cell strainer into falcon tubes and centrifuged at 300 g for 8 min at 4°C. Supernatant was discarded and each cell pellet was re-suspended in 3 ml ACK lysis buffer (Life technologies Ltd.) and incubated for 5–10 min at room temperature (RT) for red blood cell elimination. 3ml PBS was added to stop the reaction and cells were centrifuged at 300 g for 6 min. Supernatant was discarded and each pellet re-suspended in 10 ml DMEM F12 with 10% (v/v) FBS, 2 mM L- Glutamine, 100U/ml Penicillin, 100 µg/ml Streptomycin and 20 ng/ml Macrophage-colony stimulating factor (M-CSF) (Sigma Aldrich Ltd.). Suspensions were plated in 10 mm polystyrene petri dishes or 6-well tissue culture plates (Fisher Scientific Ltd.) and incubated at 37°C in a humidified environment of 5% CO_2_. After 3 days in culture, 3 ml of fresh media was added to each petri dish or well. On day 7, non-adherent cells were removed, and adherent cells were considered macrophages.

#### 2.3.3 Cell Lines

The SC5 (mdx) and IMO (WT) cell lines were derived from the leg muscle of the H2Kb-tsA58 line (Morgan et al., 1994). Cells were cultured in DMEM HG supplemented with 20% FBS, 4 mM L-glutamine, 100 unit/ml penicillin, 100 μg/ml streptomycin and 20 unit/ml murine γ-interferon (Invitrogen) at 33°C, 5% CO_2_ humidified atmosphere (Yeung et al., 2006).

RAW 264.7 cells were maintained in DMEM HG with 10% FBS, 2 mM L-glutamine, 100 unit/mL penicillin and 100 µg/ml streptomycin at 37°C and 5% CO_2_.

Cells were passaged and counted using a C-CHIP haemocytometer with trypan blue. All cells were plated at 20,000 per well in 96 well plates (Sarstedt) and incubated for 24 h to allow cell attachment prior to treatment.

Cells were treated for 48 h at 37°C using a basal media consisting of KnockOut DMEM, supplemented with 1% Knockout Serum Replacement (KSR), 0.5% v/v Donor Horse Serum (DHS, Sera Labs), 2 mM L-glutamine, 100 unit/mL penicillin and 100 µg/ml streptomycin. High phosphate media additionally contained 5 mM inorganic phosphate, 10 nM dexamethasone and 50 m g/ml ascorbic acid (Gartland et al., 2012). Additional treatments included were 20 µM AZT (Zidovudine), 1 µM brilliant blue G (BBG) and 350 µM BzATP.

Phosphate was quantified from serial dilutions of serum and cell conditioned media using the malachite green assay following manufacturer’s instructions on the SpectraMax i3x multi-mode microplate reader (Molecular Devices, Wokingham, United Kingdom). Phosphate measurements from conditioned media were normalised to total protein from cell lysates measured with the Bicinchoninic Acid (BCA) assay (Sigma).

Cell viability experiments were quantified using the Presto Blue assay using a POLARstar Optima plate reader (BMG labtech, Ortenberg, Germany) and SpectraMax i3x.

For Alizarin Red staining, plates were fixed with ice cold 70% ethanol for 20 min and air dried for 24 h. Plates were stained with 100µl/well of 40 mM Alizarin Red S (pH 4.2) for 20 min on an orbital shaker. Excess stain was removed and plates left to air dry. Dried plates were scanned in on an EPSON flatbed scanner at 1200dpi, prior to extraction with cetylpyridinium chloride and quantification of optical density at 560 nm (Stanford et al., 1995) on a SpectraMax i3x.

#### 2.3.4 Intracellular Ca^2+^ Measurements

Peritoneal macrophages were cultured on glass coverslips in 3.5 cm diameter dishes at 70–80% confluency in culture medium described above. After 24 h, cells were loaded with Fura-2 AM (Molecular Probes, Oregon) in culture medium for 20 min at 37°C in a 95% O_2_, 5% CO_2_ atmosphere. After two brief washes in the assay buffer (5 mM KCl, 1 mM MgCl_2_, 0.5 mM Na_2_HPO_4_, 25 mM HEPES, 130 mM NaCl, 1 mM pyruvate, 25 mM D-glucose, 2 mM CaCl_2_ pH 7.4) the coverslips were mounted in a cuvette and cells were treated with 300 µM of the P2X7 agonist BzATP. The calcium influx was measured in fluorescence spectrophotometer (F-7000 Hitachi). Fluorescence was recorded at 510 nm with excitation at 340/380 nm. Delta ratio of both signals was then calculated. Each experiment was repeated three times.

#### 2.3.5 Immunoblotting

Cells were scraped off in a minimal volume of extraction buffer composed of 1x LysisM extraction buffer (Cat. #04719956001, Roche), 1 protease inhibitor cocktail tablet, 1 phosphatase inhibitor cocktail tablets (both Roche) per 10 ml of buffer. Samples were homogenised by passing through a 25-gauge needle 20 times, centrifuged and protein concentrations of extracts determined using the bicinchoninic acid kit (Sigma-Aldrich Ltd.). 5–50 µg of protein were mixed with Laemmli sample buffer (Bio-Rad Laboratories Ltd.) supplemented with 5% (v/v) β-mercaptoethanol, heated at 95°C for 5 min and chilled on ice before being separated by electrophoresis in polyacrylamide gels. Samples were electrotransferred onto methanol-activated polyvinylidene fluoride membranes (Amersham System, GE Healthcare Life Sciences Ltd.). Membranes were incubated on a horizontal shaker for 1 h at RT in a blocking solution containing Tris-buffered saline with 0.1% Tween (ThermoFisher Scientific) and 5% w/v non-fat dried cow milk powder. Membranes were subsequently incubated with primary antibodies (P2X7 and P2X7-TAG, diluted 1:1000) in the same blocking solution overnight at 4°C. Membranes were washed 3 × 10 min in TBS-Tween, and incubated with appropriate HRP-conjugated secondary antibodies in the blocking solution for 1 h. Finally, membranes were washed 3 × 10 min and incubated with LuminataTM Forte chemiluminescence development reagent (Merck, Burlington, MA, United States) and subsequently imaged using a ChemiDoc MP system (Bio-Rad, Hertfordshire, United Kingdom).

#### 2.3.6 Immunocytofluorescence

Cells were cultured on coverslips (50–60%) in growth medium. After rinsing twice with cold PBS (w/o calcium and magnesium) the peritoneal macrophages were fixed in a 4% w/v paraformaldehyde solution (PFA) in PBS for 15 min on ice. Then cells were permeabilized using PBS with 0.1% Triton X-100 for 5 min and blocked in a 5% v/v normal goat serum (NGS, S-1000, Vector Laboratories IVD) in PBS for 1 h at RT and incubated overnight at 4°C with primary antibodies (anti-P2X7; 177003, Synaptic Systems, anti-TAG, PA1-984B, Invitrogen and marker of macrophages anti-F4/80; ab6640, Abcam 1:100 dilution) in blocking buffer. The secondary antibody (diluted 1:1000 in 5% NGS in PBS, Alexa Fluor 488 goat anti-Rabbit, Thermo Fisher Scientific and Alexa Fluor 555 goat anti-Rat, respectively) was added for 1 h RT in dark. Then cells were rinsed 3 times for 10 min each wash with agitation between each step. After staining, cells on coverslips were mounted onto microscope slides sealed in Glycergel Mounting Medium with DAPI (H-1200 VectaShield^®^, Vector Laboratories) prior to imaging. Images were obtained using a confocal microscope (Zeiss Spinning Disk Confocal Microscope) and image analysis was performed by using ImageJ software.

### 2.4 X-Ray Micro-computed Tomography and Analysis

Animals were harvested at 8 weeks of age. The left quadriceps muscle was fixed in 10% formalin for 48 h, then to transferred to 70% ethanol prior to scanning. Quadriceps were placed within a 2-ml tube (Eppendorf, Stevenage, United Kingdom) and supported by a polyurethane foam saturated in 70% ethanol. Muscles were imaged using a Zeiss Xradia 520 Versa X-ray microscope (Zeiss) operating at an energy of 50 kV, a power of 4 W, and a tube current of 80 μA with a Zeiss LE1 filter was positioned directly after the X-ray source. The sample distance to the x-ray source was 200mm, and the sample distance to detector was 225mm, A total of 1601 X-ray projection images were collected over 360° at equal intervals with an exposure time for each projection of 3 s. The projections were reconstructed using the manufacturer’s integrated software, which uses a filtered back projection reconstruction algorithm. TXRM files were imported into FiJi with the plugin to read XRM files and export Z-stack images (Schindelin et al., 2012; Metscher 2020). Mineralised areas within exported z-stack images (pixel size ranging from 32.25 to 34.45 µM depending upon sample) were quantified using the threshold function in ImageJ and used to calculate the volume.

### 2.5 Statistical Analysis

Statistical analyses were carried out with GraphPad Prism 9 and IBM SPSS Statistics 27. In GraphPad Prism, unpaired t-tests were used to compare two normally distributed data sets. Mann-Whitney tests were used for the comparison of two non-normally distributed data sets. Where there were three non-normally distributed data sets, a Kruskal-Wallis test was carried out with Tukey’s or Dunn’s multiple comparison tests. Where specific sets of columns required comparison, a Holm-Šídák test was used. Outliers were identified with ROUT tests. All figures containing data were made with GraphPad Prism 9. Where data was pooled from multiple replicate experiments, a Univariate analysis was carried out with Tukey’s and Dunnett’s post-tests using IBM SPSS Statistics 27.

## 3 Results

### 3.1 Elevated P2X7 Expression and Function in the Dystrophic *Dmd* ^*mdx*-βGeo^ Mouse Cells and Tissues

**FIGURE 1:**
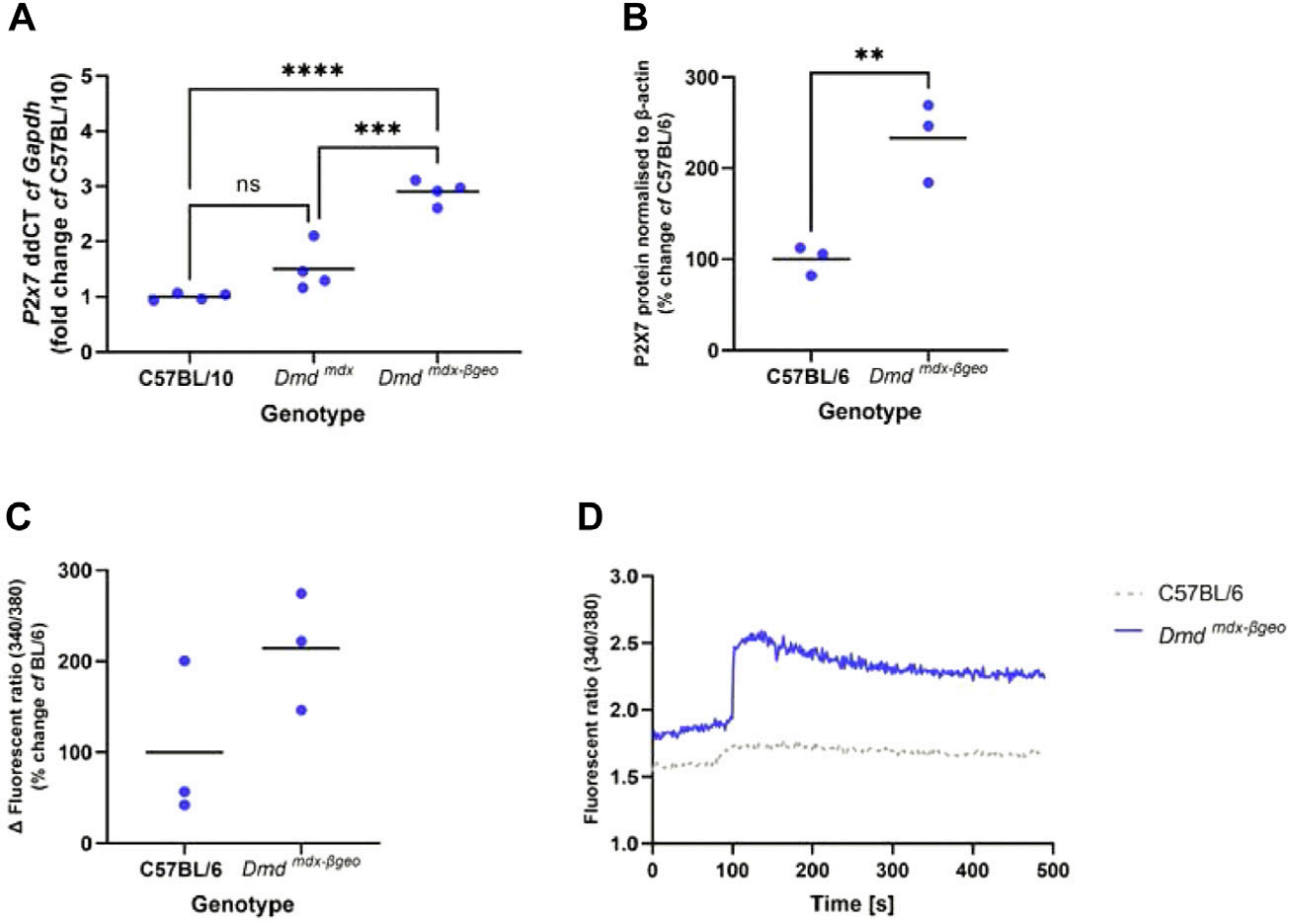
P2X7 receptor expression and function in *Dmd* ^*mdx-*βGeo^ tissues. **(A)** qPCR analysis of *P2x7* gene expression in tibialis anterior muscle from *Dmd* ^*mdx-*βGeo^ mice compared to *Dmd ^mdx^* (*** = *p* < 0.001) and wild type (**** = *p* < 0.0001), ANOVA with Tukey's post tests. **(B).** Western blot analysis of P2X7 protein expression in mouse BMM showing twice as much P2X7 in *Dmd* ^*mdx-*βGeo^ mice compared to C57BL/6 controls (** = *p* <0.01, unpaired t-test). **(C).** P2X7 activation was measured with the calcium influx assay, showing increased P2X7 activity in *Dmd* ^*mdx-*βGeo^ peritoneal macrophages. **(D).** Representative traces showing clear differences in P2X7 activation in C57BL/6 and *Dmd* ^*mdx-*βgeo^ macrophages.

We have previously demonstrated that the *Dmd* ^*mdx-*βgeo^ dystrophin-null mouse muscle presents with significantly increased ectopic myofibre calcification and altered macrophage infiltration patterns, particularly the close association of macrophages with calcified fibres (Young et al., 2020). Otherwise, the ectopic calcification had the same temporal pattern of presentation and resolution in *Dmd* ^*mdx-*βgeo^ as in the *Dmd* ^*mdx*^ mice, the most widely used animal model of DMD (Massopust et al., 2020). Therefore, we used the *Dmd* ^*mdx-*βgeo^ mouse to investigate whether the P2X7 receptor, being the key mediator of the inflammatory response, could be linked to ectopic mineral formation. Firstly, we compared *P2x7* mRNA expression in *Dmd* ^*mdx-*βgeo^ and control mouse *Tibialis anterior* muscle and found that receptor expression in *Dmd* ^*mdx-*βgeo^ muscle is significantly elevated compared to WT muscle (Figure 1A, *p* <0.001 *cf Dmd* ^*mdx*^, *p* <0.0001 *cf* C57BL/10, ANOVA with Tukey's post tests). As this receptor expression in dystrophic muscle may result from its overexpression in muscle cells or from infiltrating inflammatory cells, we compared P2X7 expression and function between bone marrow derived macrophages (BMM) isolated from wild-type C57BL/6 and *Dmd* ^*mdx-*βgeo^ mice. Using this approach, we compared naïve dystrophic and WT macrophages that were not exposed to any inflammatory signals present in the dystrophic mouse due to the chronic muscle inflammation. Such signals could confound the expression data because of the priming effect in these cells on their subsequent reactivity (Netea et al., 2016). P2X7 protein levels quantified by Western blotting were 130% higher in *Dmd* ^*mdx-*βgeo^ cells compared to C57BL/6 controls (Figure 1B, *p* < 0.01, unpaired t-test; Supplementary Figure S1). The functional P2X7 response evaluated using the intracellular calcium measurements assay showed that calcium influx after BzATP treatment was 114% higher in *Dmd* ^*mdx-*βgeo^ macrophages than in C57BL/6 controls (Figures 1C,D, *p* = 0.1425, unpaired t-test).

### 3.2 Generation of the Novel *P2rx7* ^KiKo^ Mouse Model

Targeting DNA constructs encoding tandem-tagged receptors and additionally flanked by *loxP* sites was used to generate the knock-in mouse strain. This part of the project was commissioned to the commercial developer (Ingenious Targeting Laboratory, United States). The N-termini of the P2X7a and P2X7k variants, that are uniquely expressed in mice (Xu et al., 2012) were tagged with AU1 and IRS tags, respectively to allow future identification of the native P2X7 receptor composition (subunit organization) and the full repertoire of its interacting partners (signalling and scaffolding proteins) by utilising the “genetic-proteomic” approach (Bienvenu et al., 2010). Furthermore, this P2X7^KiKo^ can be crossed with tissue-specific and/or conditional *Cre* recombinase expressing strains, leading to the excision of the *loxP*-flanked P2X7 coding region and generation of a cell/tissue specific and/or conditional P2X7 knockout (Supplementary Figure S2).

### 3.3 P2X7 Expression and Function in *P2rx7* ^KiKo^ Mouse Tissues

**FIGURE 2:**
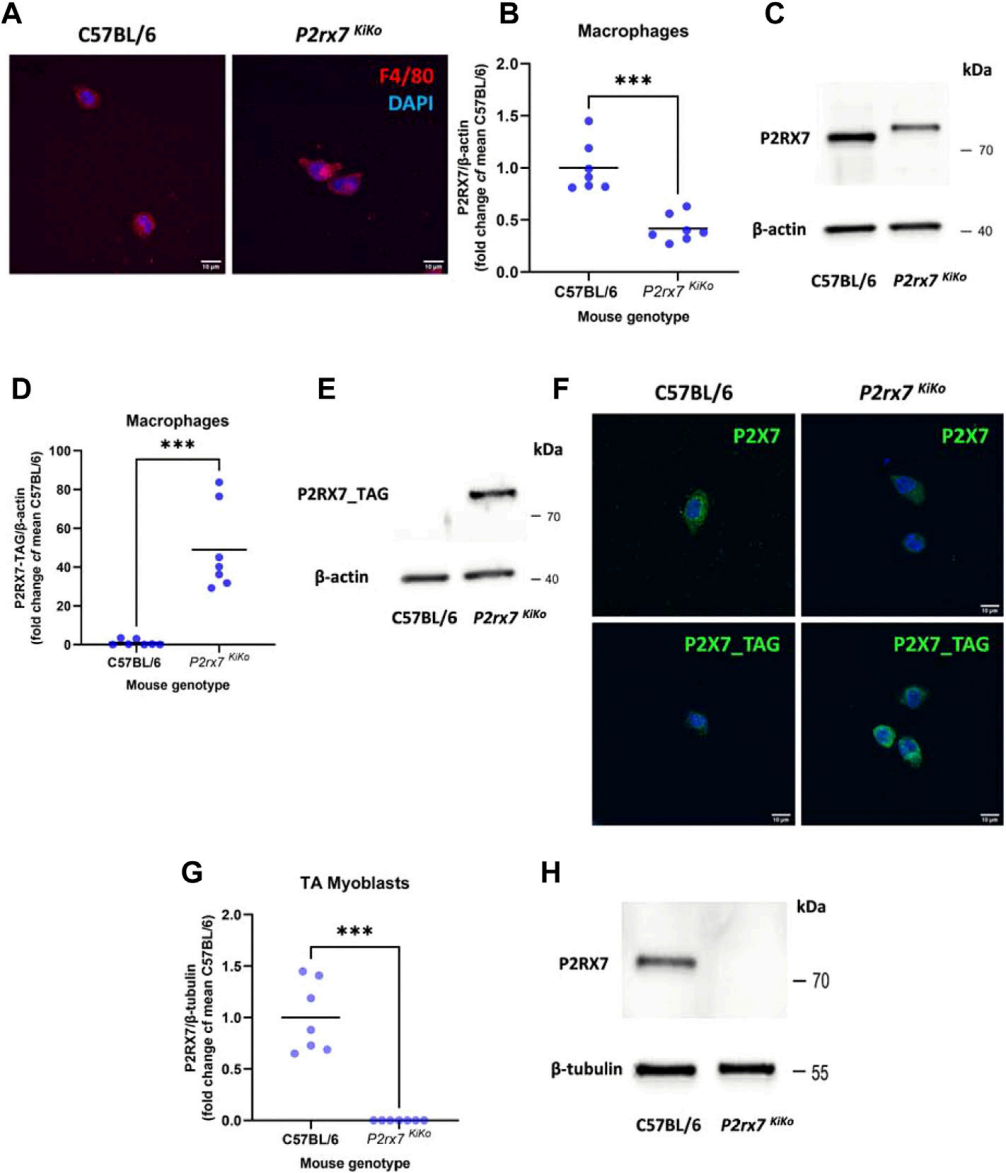
Characterisation of the *P2rx7* ^KiKo^ mouse tissues. **(A).** Immunofluorescent (IF) labelling of isolated macrophages from C57BL/6 and *P2x7* ^KiKo^ mice, using the marker F4/80 to confirm the presence of macrophages. **(B,C).** Western blot analysis of P2X7 protein expression in macrophages showing partial decrease of P2X7 tagged protein levels in *P2x7* ^KiKo^ mice compared to C57BL/6 controls (*** = *p* <0.01, unpaired t-test). **(D,E).** Western blot analysis of P2X7-TAG protein expression in macrophages demonstrating presence of P2X7-TAG in *P2x7* ^KiKo^ mice and its absence in C57BL/6 controls (*** = *p* <0.01, unpaired t-test). **(F).** IF labelling showing expression of P2X7 and P2X7-TAG in macrophages from C57BL/6 and *P2x7* ^KiKo^ mice, respectively. **(G,H).** Western blot analysis of P2X7 protein expression in myoblasts isolated from the Tibialis Anterior (TA) showing an absence of P2X7 expression in *P2x7* ^KiKo^ cells, in contrast to C57BL/6 controls (*** = *p* <0.01, unpaired t-test).

The immunodetection, molecular and functional characterisation of the resulting tagged receptor subunits in *P2rx7* ^KiKo^ mouse tissues revealed that in peritoneal macrophages the endogenous tagged P2X7 protein characterised by its slightly higher molecular weight (Figure 2C) was expressed at a slightly lower levels than the wild-type receptor but was fully functional and the responses elicited by the stimulation of the tagged P2X7 were unaffected (Figures 2A–F). In contrast, expression of the tagged P2X7 in myoblasts isolated from TA muscle was ablated, even in the absence of any *Cre* recombinase (Figures 2G,H). This unexpected outcome allowed us to use the *P2rx7* ^KiKo^ as a muscle-specific P2X7 knockout.

### 3.4 Tissue-specific P2X7 Expression Affects Ectopic Muscle Calcification

**FIGURE 3:**
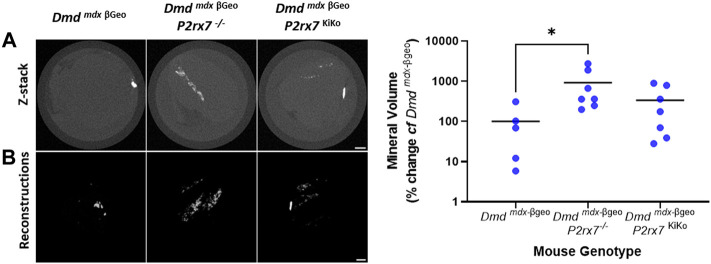
Ectopic mineralisation in *Dmd* ^*mdx-*βGeo^, *Dmd* ^*mdx-*βGeo^ *P2rx7* ^*−/−*^ and *Dmd* ^*mdx-*βGeo^ *P2rx7* ^KiKo^ quadriceps. Isolated quadriceps muscles were scanned *ex vivo* by XCT and analysed in FIJI. **(A)** Z-stack images and 3D reconstructions from representative *Dmd* ^*mdx-*βGeo^, *Dmd* ^*mdx-*βGeo^ *P2rx7* ^*−/−*^ and *Dmd* ^*mdx-*βGeo^ *P2rx7* ^KiKo^ quadriceps muscles. **(B)**. Quantification of mineralised volume within whole quadriceps muscles revealed increased mineralised volume in *Dmd* ^*mdx-*βGeo^ *P2rx7* ^*−/−*^ compared to *Dmd* ^*mdx-*βGeo^ but not *Dmd* ^*mdx-*βGeo^ *P2rx7* ^KiKo^ mice (* = *p* <0.05, Kruskal-Wallis test with Dunn’s multiple comparison test).

To investigated the role of P2X7 in ectopic calcification, *Dmd* ^*mdx-*βgeo^ mice were crossed with either global *P2rx7* knockout or *P2rx7* ^KiKo^ mice, treated as a muscle-specific knockout that retains functional P2X7 in macrophages. Quadriceps muscles isolated from 8-week-old mice were scanned by X-ray microscopy and reconstructed muscle images (Figures 3A,B) were quantified in ImageJ. There was significantly more mineral volume in quadriceps muscles from *Dmd* ^*mdx-*βgeo^ *P2rx7* ^*−/−*^ double negative mice compared to *Dmd* ^*mdx-*βgeo^ mice (Figure 3C, 926% *cf Dmd* ^*mdx-*βgeo^, *p* < 0.05, Kruskal-Wallis test, with Dunn’s multiple comparison). These calcifications were distributed throughout the muscle and did not form localised agglomerations observable in *Dmd* ^*mdx-*βgeo^ (see animations of the 3D reconstructions from representative quadriceps included in the supplementary data). In contrast, there was no significant difference in mineralised volume between *Dmd* ^*mdx-*βgeo^ and *Dmd* ^*mdx-*βgeo^ *P2rx7* ^KiKo^ quadriceps, which lack P2X7 in muscle cells (Figure 3C, 337% *cf Dmd* ^*mdx-*βgeo^, adjusted *p* value = 0.5981). These data indicate that P2X7 may have a protective effect against ectopic mineral formation in dystrophic muscle. Given that global loss of this receptor increased mineralisation but this effect was absent when P2X7 was ablated in muscle cells only, it is the loss of P2X7 in the immune cells, most likely macrophages, that is responsible for this increased calcification. Conversely, it is the expression of P2X7 in macrophages and not its overexpression in the dystrophic muscle cells that protects against ectopic calcification.

### 3.5 Phosphate Secretion is Affected by Dystrophy and Regulated by P2X7

**FIGURE 4:**
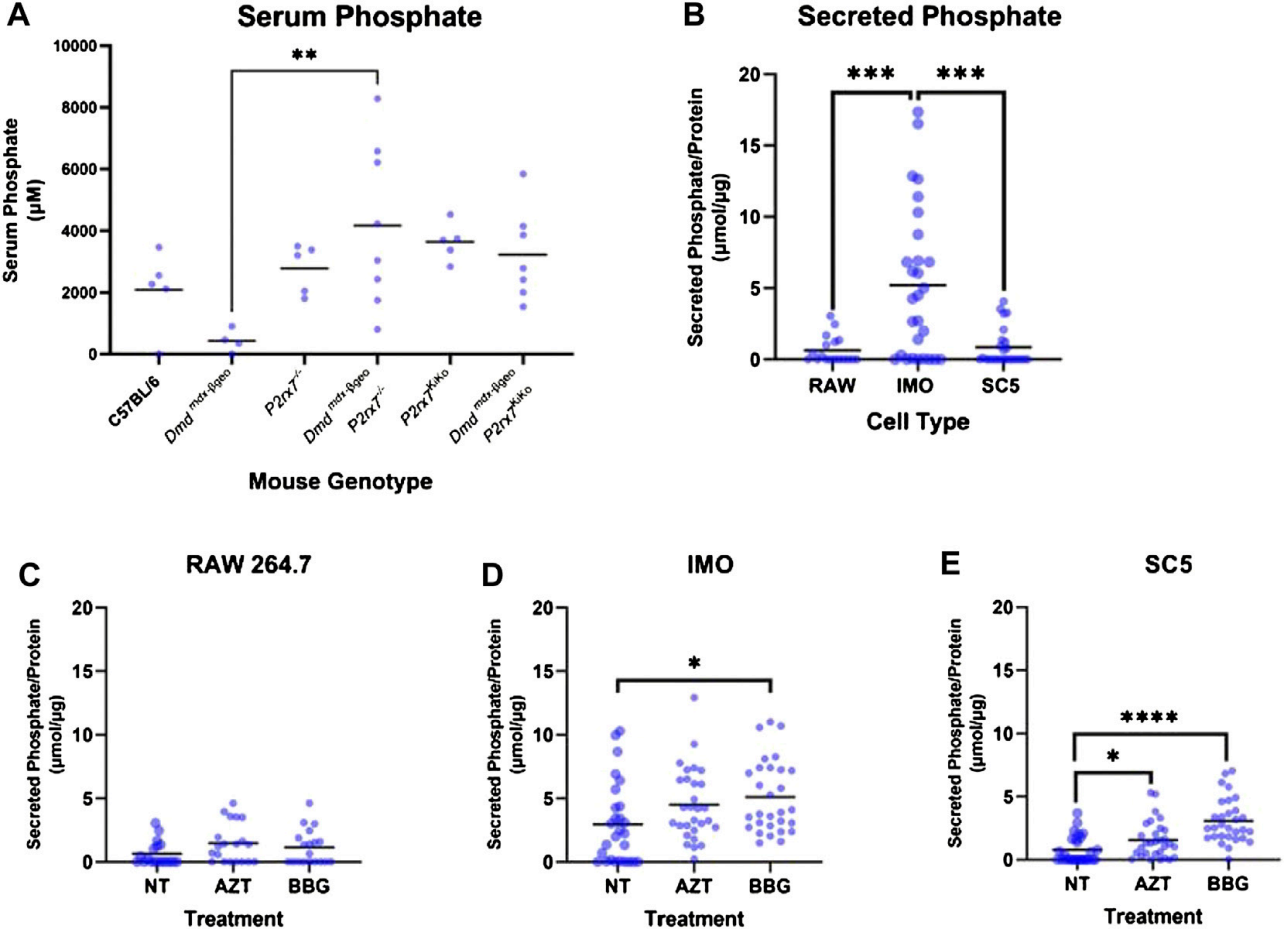
Phosphate in serum and cell conditioned media. Phosphate was measured from mouse serum and cell conditioned media using the malachite green assay. **(A)** Serum phosphate was higher in *Dmd* ^*mdx-*βGeo^ *P2rx7* ^*−/−*^ compared to *Dmd* ^*mdx-*βGeo^ but not *Dmd* ^*mdx-*βGeo^ *P2rx7* ^KiKo^ mice (** = *p* <0.01, Ordinary one-way ANOVA with Holm-Šídák’s multiple comparison test). **(B)** Phosphate levels were higher in cell conditioned media (CM) from IMO cells than from either RAW cells or SC5 cells (*p* <0.001, Univariate analysis in SPSS with Tukey’s post-tests). **(C)** Phosphate secretion by RAW cells was unaltered by treatment with the P2x7 inhibitors AZT and Coomassie brilliant blue G (BBG). **(D)** Phosphate secretion by IMO cells was increased in the presence of BBG (* = *p* <0.05, Univariate analysis in SPSS with Tukey’s post-tests). **(E)** Phosphate secretion by SC5 cells was increased in the presence of AZT (* = *p* <0.05) and BBG (**** = *p* <0.0001, Univariate analysis in SPSS with Tukey’s post-tests).

To further investigate the underlying mechanism of ectopic mineralisation, phosphate was quantified in serum samples from 8-week-old C57BL/6, *Dmd* ^*mdx-*βgeo^, *P2rx7* ^*−/−*^, *Dmd* ^*mdx-*βgeo^ *P2rx7* ^*−/−*^, *P2rx7* ^KiKo^, and *Dmd* ^*mdx-*βgeo^ *P2rx7* ^KiKo^ mice. Serum phosphate values were significantly increased in *Dmd* ^*mdx-*βgeo^ *P2rx7* ^*−/−*^ compared to *Dmd* ^*mdx-*βgeo^ mice (Figure 4A, *p* < 0.01, Ordinary one-way ANOVA with Holm-Šídák’s multiple comparisons test).

To better understand the cellular origins of these changes in serum phosphate across mouse genotypes, phosphate secretion was measured by analysing cell-conditioned media from RAW 264.7 monocytes, IMO non-dystrophic and SC5 dystrophic myoblast cell lines (Figure 4B, mean values of 0.64 μmol/μg, 5.2 μmol/μg and 0.85 μmol/μg normalised to total protein from cell lysates, respectively). This analysis demonstrated that control IMO myoblasts secreted more phosphate than dystrophic SC5 cells (*p* < 0.001, univariate analysis with Tukey post-tests). The RAW 264.7 monocytes secreted phosphate at levels comparable to SC5 cells.

As loss of P2X7 *in vivo* was associated with significantly altered serum phosphate, cells *in vitro* were treated with the P2X7 antagonist BBG and also with AZT, identified by us as an effective P2X7 blocker with good potential for repurposing for therapeutic applications (Al-Khalidi et al., 2018). Secreted phosphate levels were normalised to total protein contents in cell lysates. Phosphate secretion from RAW 264.7 cells was unaltered by P2X7 antagonists (Figure 4C). In contrast, phosphate secretion from IMO and SC5 cells was increased by treatment with BBG and AZT (Figures 4D,E, *p* < 0.0001 and *p* < 0.01 respectively, univariate analysis with Dunn’s post-tests). Interestingly, the phosphate secretion from IMO control myoblasts, which was higher than from SC5, was only mildly augmented by BBG (Figure 4D).

These data demonstrate that myoblasts are a source of secreted phosphate, and that phosphate secretion from these cells is affected by the loss of dystrophin expression. Moreover, phosphate secretion from dystrophic myoblasts is regulated by P2X7. Its increase in response to P2X7 blockade demonstrates that P2X7 overexpression on dystrophic cells (Yeung et al., 2006) may constitute a protective mechanism.

### 3.6 Induced Mineralisation of RAW 264.7, IMO and SC5 Cells is Regulated by P2X7

**FIGURE 5:**
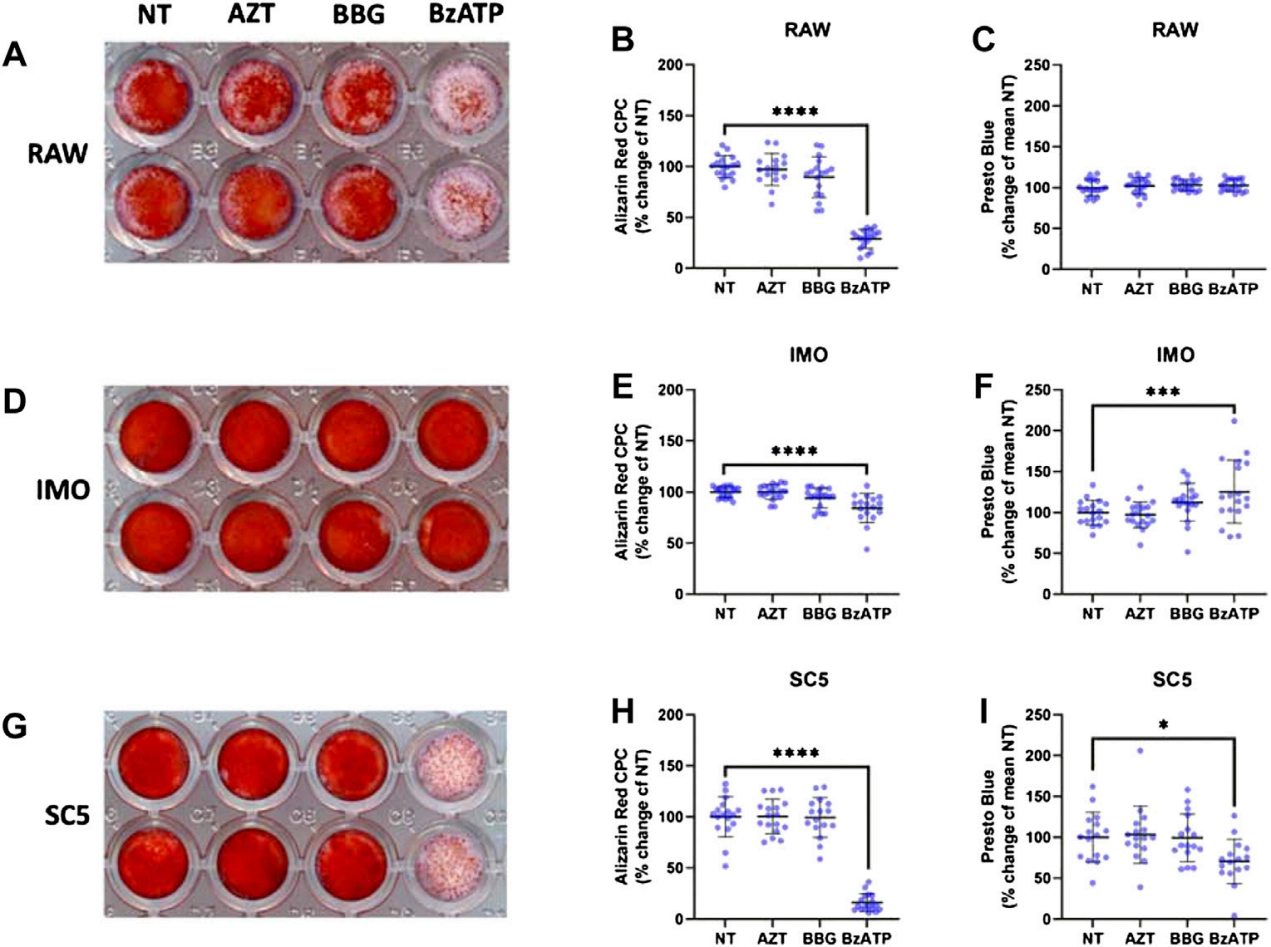
Role of P2X7 in mineralisation of RAW 264.7, IMO and SC5 cells. Cultures of RAW 264.7, IMO and SC5 cells were treated with high phosphate (HP) media, in combination with the P2X7 inhibitors AZT and BBG and the agonist BzATP for 48 h. Cell viability was measured with the Presto Blue assay and mineralisation visualised with Alizarin Red staining that was quantified with the CPC method. **(A,B)** Mineralisation of RAW 264.7 cells in HP media was decreased by the P2X7 agonist BzATP (**** = *p* < 0.0001, univariate analysis with Dunnett’s post-tests in SPSS). **(C)** Viability of RAW 264.7 cells remained unaltered across treatment groups. **(D,E)** Mineralisation of IMO cells in HP media is decreased by the P2X7 agonist BzATP (**** = *p* < 0.0001, univariate analysis with Dunnett’s post-tests in SPSS). **(F)** Viability of IMO cells was increased by the P2X7 agonist BzATP (*** = *p* < 0.0001, univariate analysis with Dunnett’s post-tests in SPSS). **(G,H).** Mineralisation of SC5 cells in HP media was decreased by the P2X7 agonist BzATP (**** = *p* < 0.0001, univariate analysis with Dunnett’s post-tests in SPSS). **(I).** Viability of SC5 cells was decreased by the P2X7 agonist BzATP (*** = *p* < 0.0001, univariate analysis with Dunnett’s post-tests in SPSS).

As macrophages are localised to regions of ectopic mineralisation *in vivo* but our data show that muscle cells act as a source of secreted phosphate *in vitro*, in the next step we investigated whether the addition of phosphate to RAW 264.7 monocyte cells in culture could engender mineralisation *in vitro* in a P2X7-dependent manner. Alizarin red (AR) staining quantified using the CPC method, revealed that RAW 264.7 cells in high phosphate media can accumulate calcified mineral deposits. In these cultures, treatment with the P2X7 agonist BzATP decreased these deposits by 71% (Figures 5A,B, *p* < 0.0001 univariate analysis with Dunn’s post-tests). Similar to phosphate secretion, mineralisation in RAW 264.7 cells was unaltered by P2X7 antagonists (Figures 5A,B). To ensure that changes in mineralisation were not connected to loss of cells or cell viability, which may occur with P2X7 activation, cells were assayed using Presto Blue prior to fixation for AR staining. There were no changes to RAW 264.7 cell viability in response to any of the treatments used (Figure 5C).

To examine the role of dystrophin in mineralisation of myoblasts, IMO control myoblasts were treated in the same manner and stained with AR (Figure 5D). CPC quantification revealed a 16% decrease in mineralisation with BzATP treatment (Figure 5E, *p* < 0.0001, Univariate analysis with Dunn’s post-tests), while cell viability quantified with Presto Blue showed an increase of ∼ 25% (Figure 5F, *p* < 0.0001, Univariate analysis with Dunn’s post-tests). Similar to the effects on phosphate secretion, P2X7 blockers did not evoke significant effect on mineralisation in IMO cells.

In SC5 dystrophic myoblast cells treated and analysed for AR, there was an 84% decrease in mineral accumulation in response to BzATP (Figure 5G, *p* < 0.0001, Univariate analysis with Dunn’s post-tests). However, in stark contrast to IMO, SC5 cell viability decreased by 30% in response to BzATP treatment (Figure 5H, *p* <0.0001).

These data show that mineral deposits can form in cultures of both monocytes and myoblasts in high phosphate conditions, typical of the dystrophic environment. Mineralisation in these cells is prevented by P2X7 activation. Loss of dystrophic myoblasts upon BzATP treatment is consistent with the overexpression and overactivation of P2X7 purinoceptor on dystrophic muscle cells (Young et al., 2012, 2015; Sinadinos and Al-Khalidi. 2015).

In conclusion, accumulation of mineral in the high phosphate environment can occur in both macrophages and muscle cells but myoblasts are the main source of phosphate release. Importantly, P2X7 purinoceptor overexpression regulates this process in dystrophic cells but it is the expression of this receptor in macrophages that protects against the ectopic mineralisation in dystrophic muscle *in vivo*.

## 4 Discussion

The main aim of this study was to investigate the role of the P2X7 receptor in ectopic mineralisation using the *Dmd* ^*mdx-*βgeo^ mouse model of muscular dystrophy which exhibits chronic inflammation associated with ectopic mineralisation of muscle fibres (Young et al., 2020). Prior to our investigation we had anticipated that activation of P2X7 could exacerbate ectopic mineralisation by driving additional inflammation. To our surprise, we found that P2X7 expression is actually protective against ectopic mineralisation, and from *in vivo* data using our novel *P2X7* ^*KiKo*^ mouse crossed with the *Dmd* ^*mdx-*βgeo^ model we determined that P2X7 on macrophages is protective.

To further investigate this phenomenon, we quantified secreted phosphate from serum and conditioned media and found that the overall pattern of serum phosphate across *Dmd* ^*mdx-*βgeo^ genotypes was consistent with ectopic mineralisation in the order of *Dmd* ^*mdx-*βgeo^ < *Dmd* ^*mdx-*βgeo^ *P2rx7* ^KiKo^ < *Dmd* ^*mdx-*βgeo^ *P2rx7* ^−/−^. Although serum phosphate has previously been reported to be higher in *Dmd* ^*mdx*^ mice than BL10 wild type controls (Kikkawa et al., 2009), we instead found a trend towards decreased serum phosphate from *Dmd* ^*mdx-*βgeo^ mice compared to C57BL/6 controls. RAW 264.7 cells secreted less phosphate than control IMO myoblast cells, suggesting that in non-dystrophic muscle the main source of secreted phosphate could be the myoblast, rather than circulatory monocytes or macrophages. The decrease in serum phosphate observed in *Dmd* ^*mdx-*βgeo^ mice was supported by a significant decrease in phosphate secretion from dystrophic SC5 myoblasts compared to non-dystrophic IMO myoblasts. Our data are consistent with previous studies on the *Dmd* ^*mdx*^ mouse insofar that we find that loss of dystrophin function leads to dysregulated phosphate secretion. The dissimilarities in data from previous studies on *Dmd* ^*mdx*^ mice and in the present study on *Dmd* ^*mdx-*βgeo^ mice might be attributable to the difference in dystrophin isoforms expressed in these two dystrophic models. In the *Dmd* ^*mdx*^ mouse there is a point mutation (Sicinski et al., 1989) preventing transcription of the full-length dystrophin isoforms, but not affecting expression of the shorter isoforms. In contrast, insertion in *Dmd* ^*mdx-*βgeo^ mice prevents expression of all dystrophin isoforms (Wertz and Martin Füchtbauer 1998). This previous body of research, together with the data presented in the current study, show that dystrophin influences phosphate secretion, and suggests that different dystrophin isoforms could have specific roles in phosphate metabolism.

We have previously found that mineralised volumes in the *Dmd* ^*mdx*^ mouse exhibit electron backscatter profiles consistent with tricalcium phosphate (hydroxyapatite) (Young et al., 2020). In this study, we found that when cells were grown under high phosphate conditions (to better mirror the dystrophic microenvironment), calcified mineral deposits were formed within cultures. Such calcification was preventable by P2X7 activation. The source of the phosphate could be myoblasts as our data suggest, while the calcium component could be found as a results of calcium dys-homeostasis, that is found across dystrophic cells (Róg et al., 2019; Zabłocka, Górecki, and Zabłocki 2021) and is a common feature of multiple forms of ectopic mineralisation (Proudfoot 2019).

Although our *in vivo* data demonstrated that P2X7 function on macrophages was protective against increased serum phosphate and ectopic mineralisation, we found little phosphate secretion from RAW 264.7 cells *in vitro*, even in the presence of the P2X7 antagonists AZT and BBG. As myoblasts secrete the bulk of phosphate and RAW 264.7 cells do not, then this is yet more evidence that macrophages may not cause the ectopic mineralisation. Macrophages have been implicated in extracellular matrix remodelling (Valentin et al., 2009; Wang et al., 2020) and more recently it has been suggested that they may even secret matrix vesicles that contribute towards ectopic mineralisation (Chen et al., 2016). In one recent study, phosphate was demonstrated to drive macrophages towards the M2 like phenotype, with increased secretion of pyrophosphate, a known inhibitor of mineralisation (Villa-Bellosta, Hamczyk, and André 2017). This is consistent with our data suggesting that macrophages may be protective against mineralisation in *Dmd* ^*mdx-*βgeo^ mice.

Global loss of P2X7 increased serum phosphate and ectopic mineral in *Dmd* ^*mdx-*βgeo^ mice; concordant with these *in vivo* findings, inhibition of P2X7 in IMO and SC5 cells *in vitro* increased phosphate secretion and activation of P2X7 in SC5 and RAW 264.7 cells in high phosphate media almost totally prevented mineral deposition. This helps to explain why retention of P2X7 in macrophages in the *Dmd* ^*mdx-*βgeo^ *P2rx7* ^KiKo^ model was associated with less ectopic mineralisation than that which occurred in *Dmd* ^*mdx-*βgeo^ *P2rx7* ^−/−^ mice, as P2X7 activation in RAW 264.7 cells is associated with decreased mineral deposition. These data suggest that in *Dmd* ^*mdx-*βgeo^ mice overall dysregulation of calcium homeostasis, coupled with phosphate secretion by myoblasts may be responsible for the initial ectopic mineralisation around both myoblasts and macrophages, but overall the role of macrophages is to protect against such ectopic mineralisation.

## Data Availability

The original contributions presented in the study are included in the article/Supplementary Material, further inquiries can be directed to the corresponding author.
