# Supplementary material for: P2X7 Purinoceptor Affects Ectopic Calcification of Dystrophic Muscles
Source: Front Pharmacol. 2022 Jul 14;13:935804. doi: 10.3389/fphar.2022.935804 (PMC9333129; doi:10.3389/fphar.2022.935804)
Supplement: Supplementary file 3 [file Presentation1.pdf]

Supplementary files

Supplementary Table 1: List of primers and used for genotyping and SYBRGreen qPCR analysis.
Genotyping primers

Target	Primer sequence
mdxβGeoGL-105 Forward (WT)	5'-GTTGAAAAGGTGAGGGCAAA-3'
mdxβGeo Rosa Beta-geo Forward (mdxβGeo)	5'-GATATCCTGTTTGGCCCA-3'
mdxβGeoGL-105 Reverse (WT and mdxβGeo)	5'-GCACGAGCATATGGTTGACA-3'
P2x7 Forward (wild type)	5'-TCACCACCTCCAAGCTCTTC-3'
P2x7 Forward (P2x7 knockout)	5'-GCCAGAGGCCACTTGTGTAG-3'
P2x7 Reverse (WT and knockout)	5'- TATACTGCCCCTCGGTCTTG-3'
P2x7 KiKo Forward	5'- TCAGCCCGGGTTCAGAAGGG-3'
P2x7 KiKo Reverse	5'- AAGTGCCCTACCTCCCCAGG-3'

Primer used for qPCR analysis

Gene symbol	Forward primer sequence	Reverse primer sequence	Amplicon size
P2rx7	5'- GGACAGCCCGAGTTGGT-3'	5'- GCTTGGAGGTGGTGATGC-3'	122
Gapdh	5'-TCAAGCTCATTTCCTGGTATGAC-3'	5'-CTTGCTCAGTGTCCTTGCTG-3'	127

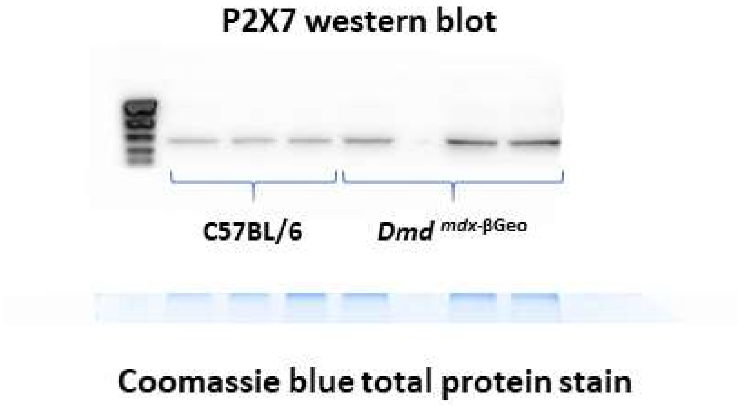

Supplementary Figure 1: Western blot analysis of P2X7 purinoceptor expression

The Western blot comparison of P2X7 protein levels in dystrophic (*Dmd*$^{mdx\text{-}\beta geo}$) and wild type (C57BL/6) bone marrow derived macrophages (BMM) used for protein quantification presented in Figure 1.

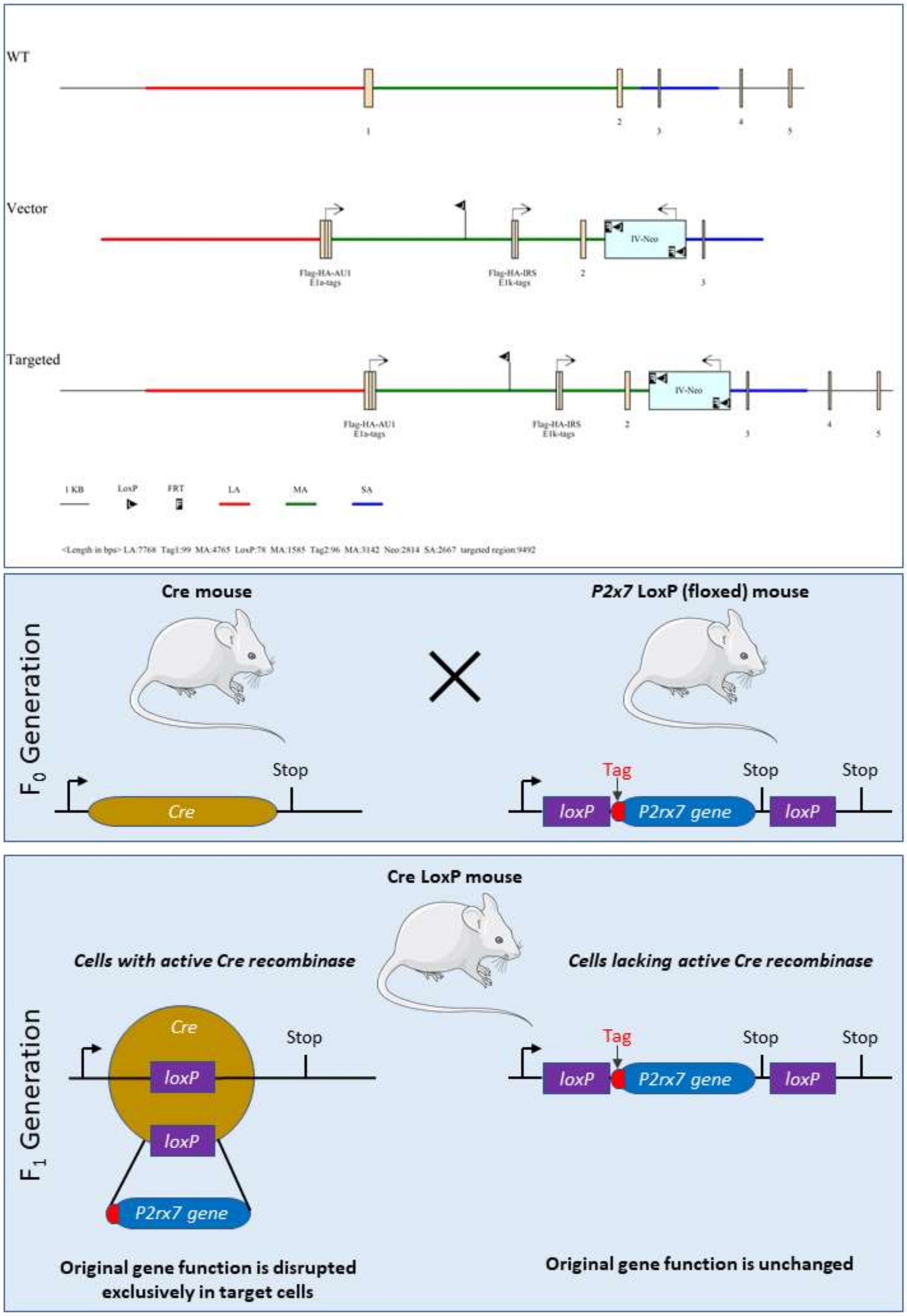

Supplementary Figure 2: Development of the *P2rx7* ^{KiKo} mouse.

Top: Schematic diagram showing the targeting strategy used in the development of the *P2rx7* ^{KiKo} mouse.

Bottom: Principles of tissue-specific ablation of floxed *P2rx7* receptor gene region. Modified from M Zepper, Wikimedia Commons contributors, "File:CreLoxP experiment.png," Wikimedia Commons, the free media repository:

ttps://commons.wikimedia.org/w/index.php?title=File:CreLoxP_experiment.png&oldid=452285805 (accessed April 25, 2022) under Creative Commons (Attribution-Share Alike 3.0 Unported, 2.5 Generic, 2.0 Generic and 1.0 Generic license) using Servier Medical Art (Creative Commons Attribution 3.0 Unported License).

Supplementary movies

Animations showing 3D reconstructions based on X-ray micro computed tomography of representative quadricep muscles from each genotype containing the *Dmd* $^{mdx\text{-}\beta Geo}$ allele.
